# Synergistic Protective Activity of Tumor-Specific Epitopes Engineered in Bacterial Outer Membrane Vesicles

**DOI:** 10.3389/fonc.2017.00253

**Published:** 2017-11-07

**Authors:** Alberto Grandi, Michele Tomasi, Ilaria Zanella, Luisa Ganfini, Elena Caproni, Laura Fantappiè, Carmela Irene, Luca Frattini, Samine J. Isaac, Enrico König, Francesca Zerbini, Simona Tavarini, Chiara Sammicheli, Fabiola Giusti, Ilaria Ferlenghi, Matteo Parri, Guido Grandi

**Affiliations:** ^1^Toscana Life Sciences, Siena, Italy; ^2^Centre for Integrative Biology (CIBIO), University of Trento, Trento, Italy; ^3^GlaxoSmithKline Vaccines (GSK) Vaccines, Siena, Italy

**Keywords:** bacterial outer membrane vesicles, cancer immunotherapy, EGRRvIII, cancer neoepitopes, BALB/c-CT26 cancer mouse model, precision medicine

## Abstract

**Introduction:**

Bacterial outer membrane vesicles (OMVs) are naturally produced by all Gram-negative bacteria and, thanks to their plasticity and unique adjuvanticity, are emerging as an attractive vaccine platform. To test the applicability of OMVs in cancer immunotherapy, we decorated them with either one or two protective epitopes present in the B16F10EGFRvIII cell line and tested the protective activity of OMV immunization in C57BL/6 mice challenged with B16F10EGFRvIII.

**Materials and methods:**

The 14 amino acid B cell epitope of human epidermal growth factor receptor variant III (EGFRvIII) and the mutation-derived CD4+ T cell neo-epitope of *kif18b* gene (B16-M30) were used to decorate OMVs either alone or in combination. C57BL/6 were immunized with the OMVs and then challenged with B16F10EGFRvIII cells. Immunogenicity and protective activity was followed by measuring anti-EGFRvIII antibodies, M30-specific T cells, tumor-infiltrating cell population, and tumor growth.

**Results:**

Immunization with engineered EGFRvIII-OMVs induced a strong inhibition of tumor growth after B16F10EGFRvIII challenge. Furthermore, mice immunized with engineered OMVs carrying both EGFRvIII and M30 epitopes were completely protected from tumor challenge. Immunization was accompanied by induction of high anti-EGFRvIII antibody titers, M30-specific T cells, and infiltration of CD4+ and CD8+ T cells at the tumor site.

**Conclusion:**

OMVs can be decorated with tumor antigens and can elicit antigen-specific, protective antitumor responses in immunocompetent mice. The synergistic protective activity of multiple epitopes simultaneously administered with OMVs makes the OMV platform particularly attractive for cancer immunotherapy.

## Introduction

All cancer therapies attempt to exploit the differences existing between tumor and normal cells. Since our immune system is built to target and destroy the “non-self,” theoretically cancer vaccination is the safest, most natural, and effective therapeutic approach against cancer. Indeed, a large number of preclinical and clinical studies involving cancer vaccines have been described over the last two decades. Unfortunately, in the clinical settings, the results so far have been disappointing. Klebanoff et al. (1) reported a cumulative analysis of several vaccine trials run from 2004 to 2009 and included 936 patients with different types of solid tumors. Using response rate as a measure of positive outcome, the conclusion of the study was that only 3.6% of the patients had an objective benefit from vaccination. The authors concluded that for cancer vaccines to become effective the strategies so far used for their formulation need be substantially revisited.

An ideal cancer vaccine should include three elements: (1) a cocktail of tumor-specific and/or tumor-associated antigens (TSA/TAAs), (2) one or more potent immune-stimulatory molecules (adjuvants), and (3) a delivery system which allows the co-delivery of cancer antigens and adjuvant(s) to antigen presenting cells (APCs). The absence of just one of these elements can make the vaccine incapable of counteracting the corrupted tumor microenvironment (containing regulatory T cells and aberrantly matured myeloid cells), and the highly mutable tumor targets (driving antigen loss and immune evasion).

Enthusiasm for therapeutic cancer vaccines has been recently rejuvenated by two major discoveries. First, it has been shown that the large number of mutations occurring in most tumors (2) creates “neo-epitopes,” which can become the targets of both CD4+ and CD8+ T cells. Neo-epitope-specific T cells have been found among tumor-infiltrating lymphocytes (TILs), and when amplified *ex vivo* from tumor biopsies and introduced back into patients, TILs can exert antitumor activities (3). Moreover, the impressive therapeutic effect of checkpoint inhibitor antibodies observed in a fraction of patients has been shown to correlate with the number of tumor-associated mutations (4–6). Consequently, vaccines formulated with neo-epitopes have recently been created and shown to be highly effective in preventing tumor growth in different preclinical settings (7). Second, Kranz and coworkers (8) have demonstrated that when administered intravenously (i.v.) in melanoma patients, negatively charged liposomes carrying TSA encoding synthetic RNAs were efficiently taken up by splenic DCs, resulting in a potent elicitation of TAA-specific CD4+ and CD8+ T cells. Overall, these data support the hypothesis that therapeutic cancer vaccines can drive protective antitumor immune responses as long as specific TSAs/TAAs are formulated with the appropriate combination of adjuvant(s) and delivery system.

In our laboratories, we have become interested in bacterial outer membrane vesicles (OMVs) both from a scientific and translation viewpoint. More than 40 years ago, researchers made the observation that all Gram-negative bacteria release OMVs, closed spheroid particles, 20–300 nm in diameter, generated through the “budding out” of the outer membrane (9, 10). Consistent with their origin, the majority of OMV components are represented by lipopolysaccharide (LPS), glycerophospholipids, and outer membrane and periplasmic proteins (11, 12). OMVs have a multitude of functions, including inter and intra species cell-to-cell cross-talk, biofilm formation, genetic transformation, defense against host immune responses, and toxin and virulence factor delivery to host cells (11). From a translational standpoint, OMVs can be an attractive vaccine platform for three main reasons. First, they carry many microbe-associated molecular patterns, including LPS, lipoproteins, peptidoglycan, and flagellin, which by binding to pathogen recognition receptors play a key role in stimulating innate immunity and promoting adaptive immune responses (13–15). Such stimulatory molecules can work synergistically, thus potentiating the built-in adjuvanticity of OMVs (16). Second, OMVs can be easily decorated with foreign antigens/epitopes by manipulating the OMV-producing strain through different Synthetic Biology approaches. This feature was demonstrated for the first time by Kesty and Kuehn who showed that *Yersinia enterocolitica* outer membrane protein Ail assembled on OMV surface when expressed in *Escherichia coli*, and that the Green Fluorescence Protein fused to the “twin arginine transport” signal sequence was incorporated in the OMV lumen (17). Following this observation, an increasing number of heterologous proteins have been successfully delivered to OMVs using a variety of strategies (16, 18). Recently, we showed that different bacterial antigens could be delivered to the lumen of *E. coli* vesicles by fusing their coding sequences to a leader peptide for secretion (19). Moreover, we showed that heterologous lipoproteins could be incorporated into the OMV membrane and that such proteins could serve as chaperones to transport heterologous polypeptides to the OMV surface (20). Third, OMVs can be rapidly and easily purified from bacterial culture supernatant. The original OMV production methods, currently in use at industrial scale for *Neisseria meningitidis* group B vaccines, involve the treatment of bacterial biomass with mild detergents (21). More recently, detergent-free methods for OMV production have been proposed which make use of mutant strains featuring a hyper-vesiculating phenotype (19, 22–25). Once the supernatant is separated from the biomass of these mutant strains, the purification of the vesicles can be easily carried out using tangential flow filtration with production yield higher than 100 mg of vesicles (protein content) per liter of culture (26).

In this work, we addressed two main questions. First, we were interested to know whether OMVs decorated with a well-known, B cell cancer-specific epitope could induce epitope-specific immune responses and whether such responses could protect immunocompetent mice from the challenge with a syngeneic cancer cell line expressing the epitope on its surface. Second, we wanted to investigate whether the addition of a second cancer-specific epitope also expressed in the same cell line could result in a synergistic effect, thus potentiating the overall efficacy of the OMV cancer vaccine. As a second epitope, we selected a protective CD4+ T cell epitope with the idea that the combination of humoral and cell-mediated immune responses could strengthen the overall anticancer effect of immunization. The data indicate that immunization with OMVs engineered with the B cell epitope strongly protected mice from tumor challenge and that 100% protection was achieved with OMVs decorated with both the B and the T cell epitopes.

## Results

### Selection of Cancer Antigens and Mouse Model

Since our first objective was to test whether the OMV-based vaccine platform could induce protective immune responses in a cancer model of immunocompetent mice, we focused our attention on C57BL/6-B16F10 model and we selected two peptide antigens, LEEKKGNYVVTDH (EGFRvIIIpep) and PSKPSFQEFVDWENVSPELNSTDQPFL (B16-M30pep), previously shown to be protective in the same model.

EGFRvIIIpep belongs to EGFRvIII, a mutated form of the human epidermal growth factor receptor (EGFR), expressed on several tumors and associated with the expression of epithelial–mesenchymal transition and cancer stem cell genes. EGFRvIII contains an in-frame deletion in the extracellular domain of EGFR, creating a novel antigenic epitope which is exquisitely tumor-specific (27). Immunization with EGFRvIIIpep conjugated to limpet hemocyanin (KLH) was shown to protect mice from the challenge of syngeneic cell lines stably transfected with human EGFRvIII. In particular, Heimberger and coworkers showed that the conjugated peptide formulated with GM-CSF protected C57BL/6 mice from both extracerebral and intracerebral challenge with B16F10-EGFRvIII cells (28). Based on these data, a vaccine (Rindopepimut) for EGFRvIII-positive glioblastoma patients was proposed and tested in different trials (29). As far as the B16-M30pep is concerned, it was recently described by Kreiter and coworkers (7) as a CD4+ T cells epitope expressed in the B16F10 cell line as a consequence of a mutation occurred in the *kif18b* gene. Therefore, M30 is a B16F10-specific neo-epitope not expressed in the syngeneic healthy C57BL/6 mouse tissues. Interestingly, the authors showed that immunization with liposome-formulated synthetic RNA coding for B16-M30 induced robust T cell-mediated protection in C57BL/6 mice when challenged with B16F10 cells.

### Immunogenicity and Protective Activity of EGFRvIII-OMVs

**Figure 1:**
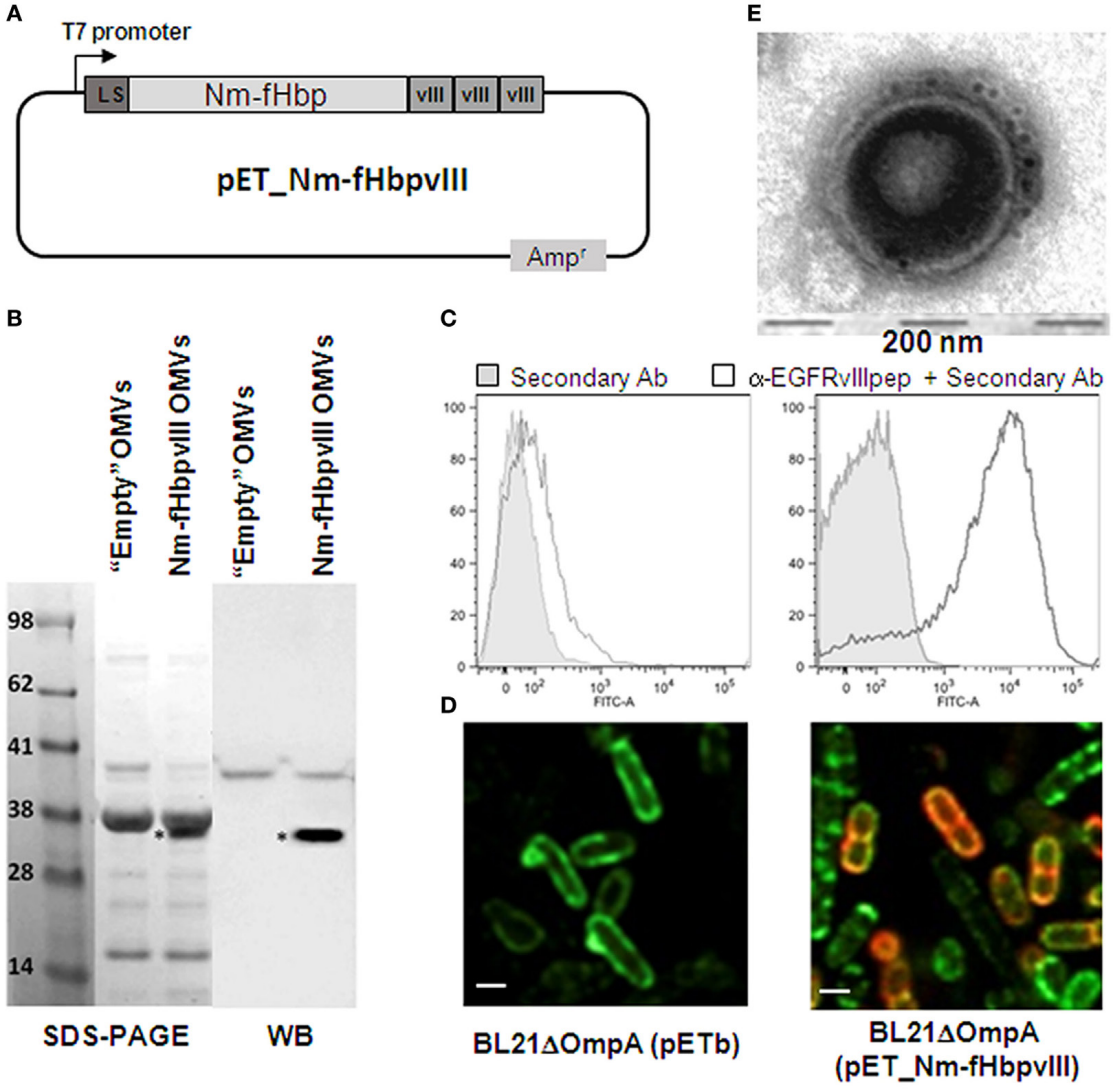
Expression and surface localization of EGFRvIII epitope in BL21*ΔompA*(pET-Nm-fHbpvIII) strain *and in its derived outer membrane vesicles (OMVs)*. **(A)** Schematic representation of pET-Nm-fHbpvIII plasmid encoding three copies of EGFRvIIIpep fused to the C-terminus of *Neisseria meningitidis* fHbp. **(B)** SDS-PAGE and Western Blot analyses of OMVs. OMVs were purified from BL21*ΔompA*(pET21b+) (“Empty” OMVs) and BL21*ΔompA*(pET-Nm-fHbpvIII) strains and loaded on SDS-polyacrylamide gels for SDS-PAGE analysis (20 µg OMVs) and Western Blot analysis (1 µg OMVs). After proteins transfer to the nitrocellulose membrane, Nm-fHbp-vIII fusion was visualized using rabbit anti-EGFRvIIIpep antibodies and peroxidase-conjugated anti-rabbit immunoglobulins. **(C)** Flow cytometry analysis of BL21*ΔompA*(pET21b+) and BL21*ΔompA*(pET-Nm-fHbpvIII) strains. Bacterial cells were incubated first with anti-EGFRvIIIIpep rabbit antibodies and subsequently with FITC-labeled anti-rabbit secondary antibodies. Fluorescence was measured by flow cytometry. Gray areas represent the background fluorescence signals obtained incubating the cells with the secondary antibody only. **(D)** Confocal microscopy analysis of BL21*ΔompA*(pET21b+) (“Empty” OMVs) and BL21*ΔompA*(pET-Nm-fHbpvIII) strains. After induction of protein expression with IPTG, bacterial cells were fixed in 4% formaldehyde solution and incubated first with rabbit anti-EGFRvIIIpep polyclonal antibodies and mouse anti-LPS mAb, and subsequently with goat anti-rabbit IgG, Alexa Fluor 594 conjugated-antibodies (red), and goat anti-mouse IgG, Alexa Fluor 488 conjugated-antibodies (green). **(E)** Immuno Transmission Electron Microscopy (TEM) analysis of OMVs purified from BL21*ΔompA*(pET-Nm-fHbpvIII) strain using primary anti-EGFRvIIIpep rabbit antibodies and 5-nm gold-labeled anti-rabbit secondary antibody (see Materials and Methods for details).

We first tested whether OMVs decorated with the Nm-fHbp-vIII fusion protein carrying three copies of EGFRvIIIpep at its C-terminus could induce anti-EGFRvIIIpep antibodies and whether such anti-EGFRvIIIpep immune response could protect mice from B16F10EGFRvIII challenge. The expression of EGFRvIIIpep in the OMVs from *E. coli* BL21*ΔompA* strain has been recently described (20). Briefly, a synthetic DNA encoding three copies of EGFRvIIIpep was fused to the 3′ end of the *Neisseria meningitidis* fHbp gene, thus generating a chimera (Nm-fHbp-vIII) constituted of the full length fHbp protein and the EGFRvIII tri-peptide attached to its C-terminus (Figure 1A). The fusion protein was shown to be incorporated into the outer membrane of *E. coli* BL21*ΔompA* and importantly to be exposed on the cell surface (Figures 1C,D). Furthermore, Nm-fHbp-vIII accumulates in the vesicle compartment, as demonstrated by SDS-PAGE and Western Blot analyses (Figure 1B) and by immune gold transmission electron microscopy (TEM) analysis of OMVs (Figure 1E).

**Figure 2:**
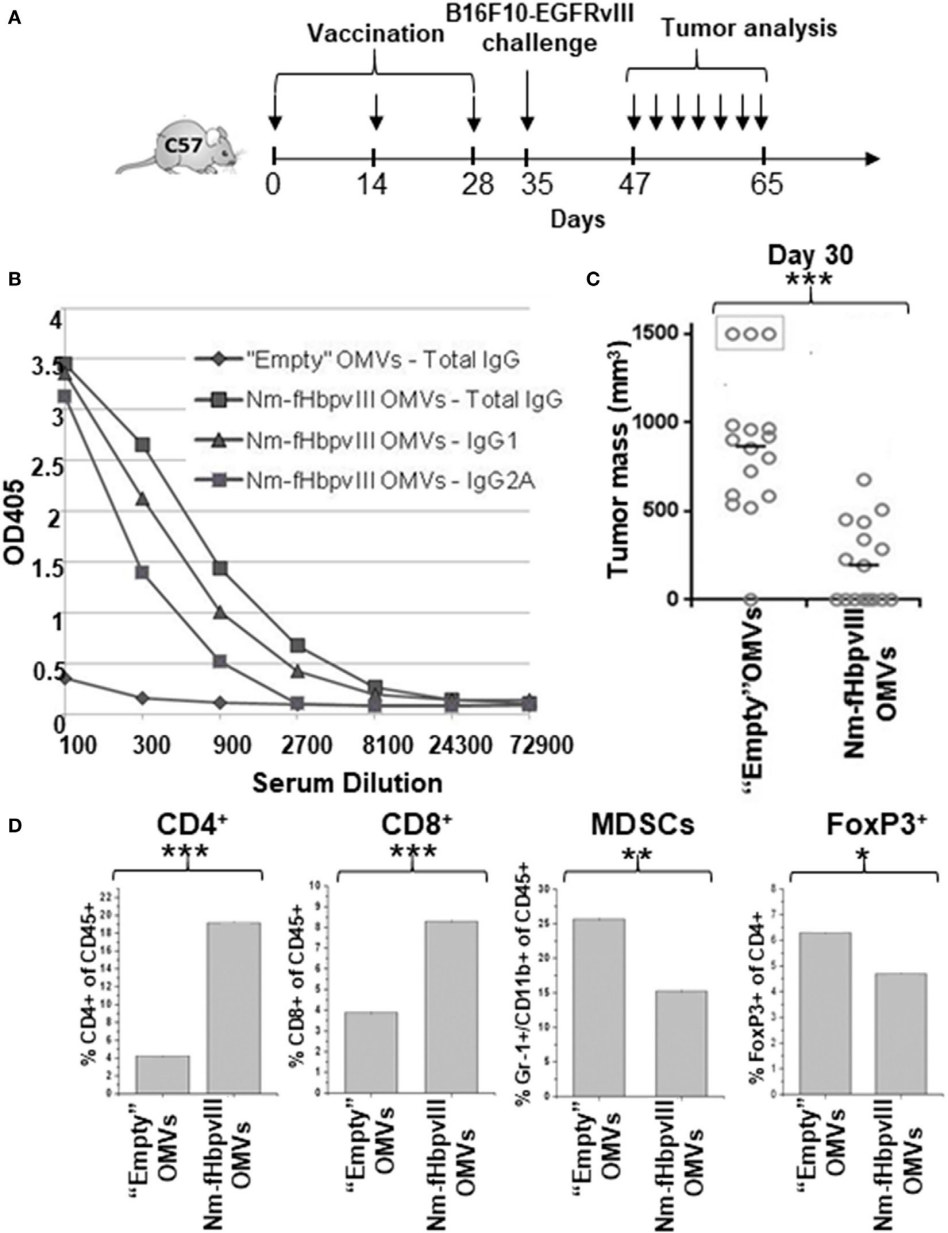
Immunogenicity and protective activity of Nm-fHbpvIII-outer membrane vesicles (OMVs). **(A)** Schematic representation of immunization and challenge schedules in C57BL/6 mice. **(B)** Anti-EGFRvIIIpep antibody titers in C57BL/6 mice immunized with “Empty” OMVs and with Nm-fHbpvIII-OMVs. Sera from mice immunized as reported in **(A)** were pooled and total IgGs, IgG1, and IgG2a were measured by ELISA, coating the plates with synthetic EGFRvIIIpep (0.5 μg/well). **(C)** Analysis of tumor development in C57BL/6 mice immunized with “Empty” OMVs and with Nm-fHbpvIII-OMVs. The figure reports the tumor size in each mouse as measured at day 30 after challenge with 0.5 × 10^5^ B16F10EGFRvIII cells. *** indicates a statistically significant difference of *P* < 0.001. **(D)** Analysis of tumor-infiltrating cell populations. At the end of the challenge experiment, two tumors/group were randomly selected and the percentage of infiltrating CD4+ T cells, CD8+ T cells, MDSCs, and Tregs was determined by flow cytometry, as described in Section “Materials and Methods” (*P < 0.05; **P < 0.01; ***P < 0.001).

C57BL/6 mice (16 mice per group) were immunized with either “empty” OMVs (not carrying the fused antigen) from *E. coli* BL21*ΔompA* (control group) or with Nm-fHbp-vIII-OMVs. Vaccination was carried out at days 0, 14, and 28 (Figure 2A) and 1 week after the third immunization sera were collected and the induction of anti-EGFRvIII-antibodies was confirmed by ELISA (Figure 2B). A good fraction of EGFRvIII-specific antibodies belonged to the IgG2a isotype, in line with our previous data showing that OMVs from *E. coli* BL21*ΔompA* elicit a Th1-skewed immune response (19). Next, at day 35, mice were challenged with a s.c. injection of 0.5 × 10^5^ B16F10EGFRvIII cells and tumor growth was followed both in control mice and in mice immunized with Nm-fHbp-vIII-OMVs. While all but one control mice developed large tumors 20 days after challenge (average tumor volume = 850 mm^3^, with three mice sacrificed having developed tumors >1,500 mm^3^), immunization with Nm-fHbp-vIII-OMVs markedly reduced tumor growth in a statistically significant manner. In particular, eight mice were completely protected while the remaining mice developed tumors with average volumes of approximately 400 mm^3^ (Figure 2C).

We also analyzed the tumor-infiltrating cell population in both control and Nm-fHbp-vIII-OMVs immunized mice. At the end of the challenge study, two tumors per group were randomly collected. Cells were mechanically and enzymatically isolated and the fraction of CD4+ T cells, CD8+ T cells, Treg, and MDSCs populations was determined by flow cytometry analysis after cell staining with specific antibodies. As shown in Figure 2D (Figure S1 in Supplementary Material), in line with the Th1 profile of the immune response, Nm-fHbp-vIII-OMVs immunization promoted a significant increase of CD4+ and CD8+ T cells at tumor site and a concomitant reduction of both CD4+ Treg and MDSC cells.

### Synergistic Protective Activity of EGFRvIIIpep and M30

**Figure 3:**
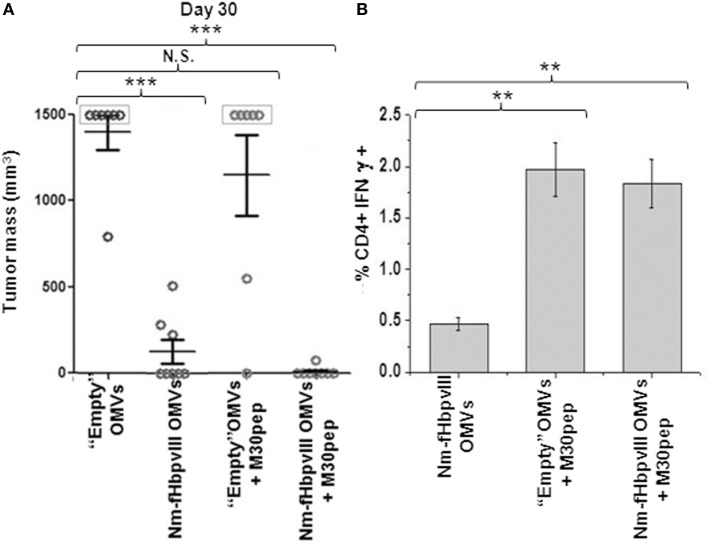
Synergistic protective activity of Nm-fHbpvIII-OMVs/M30 peptide combination. **(A)** Analysis of tumor development in C57BL/6 mice immunized as shown in Figure 2A. The figure reports the tumor size in each mouse as measured at day 30 after the challenge with 0.5 × 10^5^ B16F10EGFRvIII cells. *** indicates that the difference in tumor size between each group and control group is statistically significant with *P* < 0.001. **(B)** Analysis of M30pep-specific CD4+ T cells in immunized mice. At the end of the challenge experiment, spleens from two animals were collected. Splenocytes were stimulated with M30pep and IFNγ-producing CD4+ T cells were analyzed by flow cytometry (**P < 0.01).

Having demonstrated that EGFRvIII-OMVs induced a robust protection in C57BL/6 mice challenged with B16F10EGFRvIII cell line, we investigated whether protection could be further potentiated by formulating Nm-fHbp-OMVs with B16-M30pep, a second antigen expressed in B16F10EGFRvIII and generated by one of the several B16F10-specific mutations (7). Therefore, we set up a second immunization/challenge experiment involving four groups of eight mice each. The first group received three doses of “empty” OMVs from *E. coli* BL21*ΔompA* (control group). The second group was injected with “empty” OMVs together with B16-M30 synthetic peptide (100 μg/dose) (peptide-“absorbed” M30-OMVs). Finally, the third and the fourth groups received three doses of Nm-fHbp-vIII-OMVs and three doses of Nm-fHbp-vIII-OMVs mixed with B16-M30pep, respectively (peptide-“absorbed” M30-Nm-fHbp-vIII-OMVs). One week after the last immunization, all mice were challenged with 0.5 × 10^5^ B16F10EGFRvIII cells and tumor growth was followed over a period of 30 days. Figure 3 summarizes the result of this experiment. In line with the previous experiment, EGFRvIII-OMVs induced a strong protective immunity against B16F10EGFRvIII. Five out of eight mice were completely protected and the other three mice developed tumors with an average size of approximately 350 mm^3^. All but one control mice developed tumors ≥1,500 mm^3^ and were euthanized. As far as M30-“absorbed” vesicles are concerned, M30-OMVs immunization resulted in a marginal, non-statistically significant protection, with only two out of eight mice protected. However, when B16-M30 peptide was “absorbed” to Nm-fHbp-vIII-OMVs, protection from tumor growth was complete, with only one mouse scored as having a “barely detectable tumor” (Figure 3A).

The conclusion from these experiments is that the M30 peptide “absorbed” to “Empty” OMVs induced an M30-specific immune response not sufficient to protect mice from the challenge with B16F10EGFRvIII cell line, but capable of synergizing with a second antigen (a B cell epitope) to the point that together the two antigens completely abrogated tumor growth.

To evaluate the immunogenicity of the M30 peptide “absorbed” to OMVs, the presence of M30-specific, IFN-γ-positive T cells was analyzed in the spleens of mice sacrificed at the end of the challenge experiment. As shown in Figure 3B, mice immunized with both M30-OMVs and M30-EGFRvIII-OMVs had a higher number of M30-specific, CD4+ T cells with respect to spleens of control-group mice. By contrast, no appreciable amounts of M30-specific CD8+ T cells were measured (not shown), in line with the fact that M30 was described as a MHC II neo-epitope (7).

**Figure 4:**
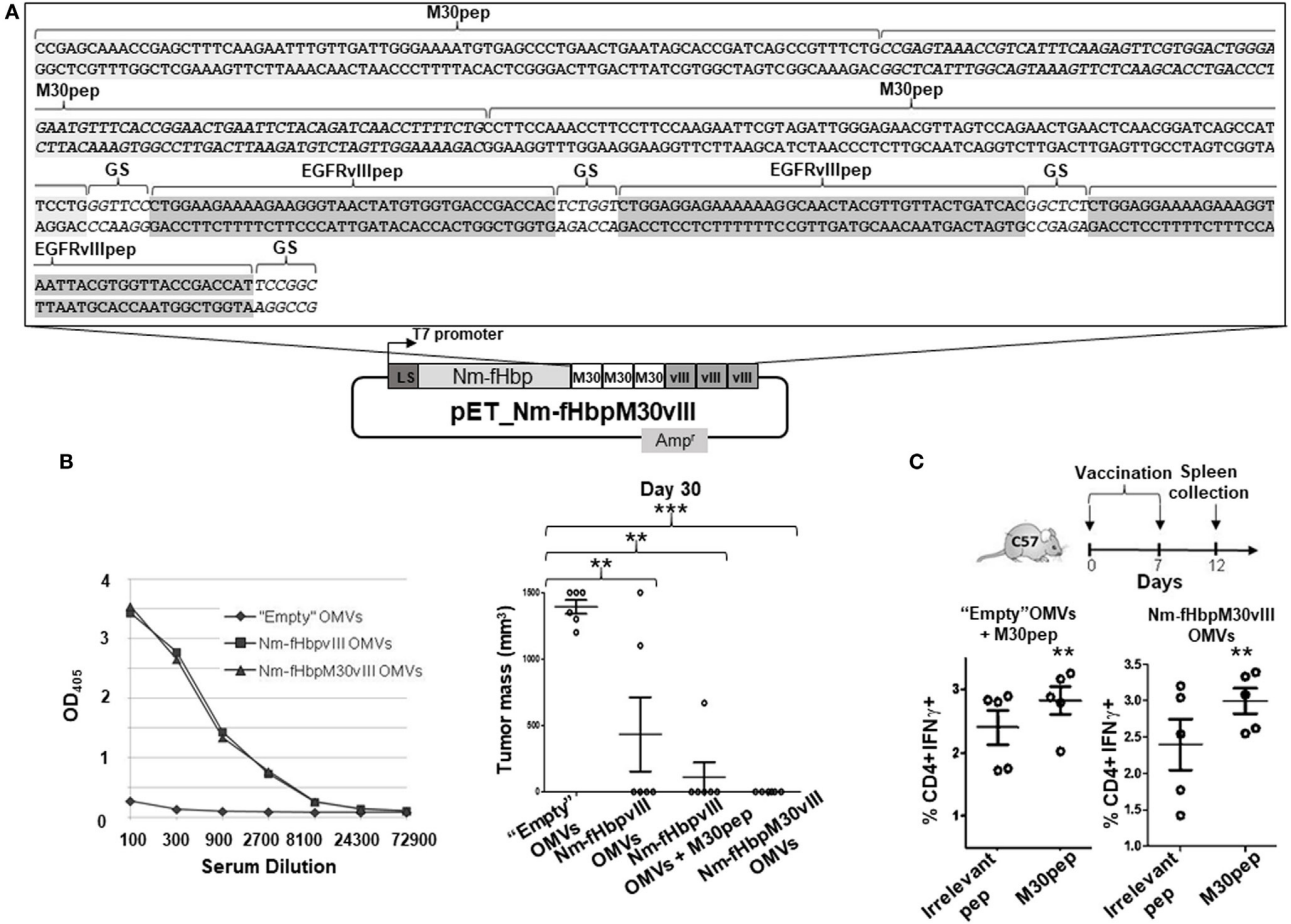
Immunogenicity and protective activity of outer membrane vesicles (OMVs) decorated with Nm-fHbp-M30-vIII fusion. **(A)** Schematic representation of pET-Nm-fHbp-M30-vIII plasmid. The DNA sequence refers to the 3′ end of the gene fusion encoding three copies of both M30 and EGFRvIII epitopes. **(B)** Mice were immunized with (i) “Empty” OMVs, (ii) Nm-fHbpvIII OMVs, and (iii) Nm-fHbpvIII OMVs + M30pep, and (iv) Nm-fHbp-M30-vIII-OMVs and subsequently challenged with 0.5 × 10^5^ B16F10EGFRvIII cells following the schedule indicated in Figure 2A. Tumor size in each mouse was measured at day 30 post challenge. Seven days after the last immunization, serum samples were also collected from mice immunized with (i) “Empty” OMVs, (ii) Nm-fHbpvIII OMVs, and (iii) Nm-fHbp-M30-vIII-OMVs, and total anti-EGFRvIII IgGs were measured by ELISA (**P < 0.01; ***P < 0.001). **(C)** M30-specific CD4+ T cells induced in mice immunized with OMVs decorated with M30 peptide. Mice were immunized twice i.p. at days 0 and 7 with either 20 µg “Empty” OMVs + 100 mg M30pep or 20 µg Nm-fHbp-M30-vIII-OMVs. Five days after the second immunization, splenocytes were stimulated with either an irrelevant peptide or with M30 peptide and IFNγ-positive CD4+ T cells were counted by FACS (**P < 0.01).

Finally, to further confirm the synergistic effect of EGFRvIIIpep and M30 in protecting mice from B16F10EGFRvIII cell line challenge, we created a second fusion protein in which fHbp was fused to three copies of M30 peptide followed by three copies of EGFRvIII pep (Figure 4A). The construction details of plasmid pET-Nm-fHbp-M30-vIII encoding the fusion protein are reported in the Section “Materials and Methods.” The fusion protein accumulated in the OMV compartment and the engineered OMVs induced anti-EGFRvIII antibody titers similar to the titers induced by Nm-fHbp-vIII-OMVs (Figure 4B). Moreover, Nm-fHbp-M30-vIII-OMVs induced IFN-γ positive, M30-specific, CD4+ T cells to a level comparable to the induction observed upon immunization with M30—“absorbed” OMVs (100 µg M30pep + 20 µg OMVs) (Figure 4C). Finally, when mice immunized with Nm-fHbp-M30-vIII-OMVs were challenge with the B16F10EGFRvIII cell line, all animals were completely protected with no sign of tumor development at the site of injection (Figure 4B).

## Discussion

This work delivers a few relevant messages.

First of all, we have shown that the OMV vaccine platform can be potentially applicable in cancer immunotherapy. OMVs are being extensively and successfully utilized in the preclinical and clinical settings for prophylactic vaccination against infectious diseases [for a recent review, see Ref. (30)]. Their unique adjuvanticity, which directs the immune responses toward a marked Th1 profile, and the ease with which they can be manipulated and purified have attracted the attention of several academic and industrial groups and bacterial OMV-based vaccines are already available for human use. However, there is a paucity of information regarding the applicability of this platform technology in cancer vaccines. Our data demonstrate that OMVs are a promising alternative to other adjuvants/delivery systems. EGFRvIII-decorated vesicles are capable of inducing a potent anti-EGFRvIII antibody response which, in immune competent C57BL/6 mice, strongly reduced the growth of B16F10 tumor cells expressing human EGFRvIII. Moreover, the Th1 profile of the response favored the migration of IFNγ-producing CD4+ and CD8+ T cells at the tumor site, eventually contributing to the overall protective activity of vaccination. The level of protection obtained appears to be similar to the one described by Heimberger and coworkers using the same mouse model and a KLM-conjugated EGFRvIII peptide in the presence of GM-CSF (28).

A second important message from this work is that the decoration of OMVs with more than one antigen further potentiate the protective efficacy of the vaccine. In particular, we combined a B cell epitope to a CD4+ T cell epitope and we showed that, together, the two epitopes completely abrogate tumor growth. This is an interesting observation also in light of the fact that in glioblastoma patients, vaccination with EGFRvIII-conjugated peptide was shown to prolong overall survival but ultimately EGFRvIII-negative tumor cells escape vaccine-induced protection (29). This immunoediting mechanism can in part explain the disappointing results obtained with the EGFRvIII-conjugated vaccine in a large Phase III trial (31). Our data pointing to the synergistic effect of EGFRvIII-OMV in combination with other cancer-specific epitopes might rejuvenate the interest in EGFRvIII antigen in the near future.

A third message from our work is the confirmation that the OMV platform can efficiently elicit not only humoral but also cell-mediated immunity against OMV-associated heterologous antigens. Even though the elicitation of protective T cell responses using pathogen-derived whole vesicles (32) or using OMVs decorated with heterologous antigens (33) was described, information on the general applicability of the OMV platform to induce antigen-specific cell-mediated immunity is still limited. Our work further provides evidence that OMVs combined or engineered with new T cell epitopes elicit epitope-specific T cell responses. We recently corroborated this conclusion by engineering OMVs with seven additional cancer CD4+/CD8+ T cell epitopes and by demonstrating the induction by all seven engineered OMVs of epitope-specific T cell responses (manuscript in preparation). Considering the ease with which OMVs can be manipulated with foreign antigens, these results lead to the attractive possibility of exploiting the OMV platform in cancer precision medicine.

One last comment deserves the strength of T cell responses induced by OMVs. Kreiter and coworkers previously shown that the M30 CD4+ T cell epitope completely inhibited tumor growth using a mouse model similar to the one tested in this study. Furthermore, the same authors showed that M30 immunization could also reduce the formation of lung metastases in the same model (7). In our hands, protection mediated by M30-“absorbed” OMV immunization could only be appreciated in combination with EGFRvIII epitope. There are a number of arguments to explain the different results. First, differently from the data reported by Kreiter and coworkers (7), we used a “classical” prophylactic modality according to which three immunizations were followed by the tumor challenge. While this schedule is indicated for eliciting antibody responses, it is not typically recommended for T cell responses, which usually require several administrations few days apart. Second, the i.v. route of immunization used by Kreiter and coworkers appears to be a key element to obtain the remarkable protection of M30 peptide. By delivering the vaccine intravenously, these authors showed that the vaccine could reach the spleen where it could be taken up by dendritic and phagocytic cells. Third, Kreiter and coworkers used as vaccine synthetic RNA coding for the M30 peptide. RNA vaccines have the property to drive the expression of the antigen directly into the cytoplasm of receiving cells and to act as potent adjuvant. While the armamentarium of adjuvants present in OMVs, which work through the elicitation of several TLR and NOD signaling pathways, should guarantee excellent Th1 immune responses, we are currently testing whether different immunization schedules and routes of immunization might improve the level of protection of M30-formulated OMVs. In this respect, we recently challenged BALB/c mice with CT26 cell line, and after challenge, mice were given seven immunizations 3 days apart with OMVs decorated with five protective CT26 neoeptopes described by Kreiter et al. (7). Following this therapeutic immunization modality tumor growth was remarkably reduced (manuscript in preparation). Finally, it has to be pointed out that when tested alone in our immunization/challenge experiments M30 peptide was “absorbed” to OMVs. In reality, we do not know the interaction of the M30 peptide to the OMVs and in fact, considering the hydrophobic nature of several amino acids and the presence of a few negatively charged amino acids, the peptide might not stably interact with the vesicles at all. Should this be the case, since adjuvant/antigen co-delivery to DCs is a pre-requisite to elicit good T cell responses, M30-engineered OMVs should outperform the M30-“absorbed” OMVs. We have not tested yet the protective activity of M30-engineered OMVs but it is interesting to note that Nm-fHbp-M30-vIII-OMVs fusion induced good levels of M30-specific, CD4+ T cells and fully protected mice from tumor challenge even if, on a molar basis, the amount of M30 peptide present in the engineered OMVs was approximately 1,000-folds lower than the 100 µg theoretically “absorbed” to the OMVs. In fact, assuming that the fusion protein represents 2–5% of total OMV proteins (Figure 4B), each mouse received approximately 0.5–1 µg of fusion protein/vaccine dose, corresponding to no more than 50–100 ng of M30 peptide.

In conclusion, our work demonstrates that bacterial OMVs represent a promising platform for cancer immunotherapy. The main interesting aspects of the technology includes (i) the rapidity with which they can be decorated with foreign epitopes (we routinely engineer the OMV-producing strains with heterologous antigens in less than 2 weeks), (ii) the high yield of OMVs from bacterial fermentation (usually more than 100 mg of purified OMVs are obtained from a 1-l fermentation), and (iii) the simplicity of the OMV purification process, which only involves tangential flow ultrafiltration. Considering that OMVs are already part of specific human vaccines for which the safety and the quality control assays have already been developed, the platform is potentially ready to be tested in the clinics.

## Materials and Methods

### Bacterial Strains, Cell Line, and Mice

*Escherichia coli* HK100 strain was used for cloning experiments using the PIPE method.

B16F10 melanoma cell line that stably expresses the EGFRvIII variant gene was kindly provided by Prof. Sampson (Department of Neurosurgery of the Duke University, Duhram, NC, USA). Cells were tested for mycoplasma before animal injection.

To verify the presence of the M30-associated mutation in B16F10 cell line, RNA from B16F10 cells was purified using RNeasy Mini Kit (Qiagen) according to manufacturer’s instructions. Subsequently, purified RNA was reverse-transcribed to cDNA using qScript cDNA synthesis kit (Quanta Bioscences). Finally, the region spanning the M30-associated mutation was PCR amplified from B16F10 cDNA with the forward (TCCTCCCGAGTCTGCCCAGCCACGGTCATT) and the reverse (ACAGCTGCGGCCTCGGGAGACTGAGGGCCT) primers. The amplification reaction product was purified from agarose gel using the PCR clean-up Kit (Macherey Nagel) and sequenced.

C57bl/6 female 4-week-old mice were purchased from Charles River Laboratories and kept and treated in accordance with the Italian policies on animal research at the Toscana Life Sciences animal facility (Siena, Italy).

### Construction of Plasmids

The construction of pET21-Nm-fHbp and pET-Nm-fHbp-vIII plasmids expressing the *Neisseria meningitidis* fHbp and fHbp fused to three repeated copies of EGFRvIII peptide, respectively, was previously described (20). pET-Nm-fHbp-M30vIII plasmid carries the *N. meningitidis fHbp* gene fused to a synthetic DNA fragment encoding three copies of B16-M30 peptide and three copies of EGFRvIII peptide, each copy intercalated by a Glycine–Serine (GS) spacer (Figure 4A). To construct the plasmid, the PIPE method was applied. Briefly, pET-Nm-fHbp-vIII plasmid was linearized by PCR, using F-vIIIM30 (5′-ATCAGCCATTCCTGGGTTCCCTGGAAGAAAAGAAGGGT-3′) primer, which anneals upstream of the vIII coding sequence, and R-fHbpM30(5′-TGCCTAGTCGGTAAGGACTTATTGCTTGGCGGCAAGGC-3′) primer. In parallel, the synthetic DNA encoding three copies of M30 peptide (Thermo Fisher, 1 ng/µl in MilliQ water) was amplified by PCR with the forward primer 5′-CTTGCCGCCAAGCAACCGAGCAAACCGAGCT-3′, complementary to the 5′ end of the *N. meningitidis fHbp* gene, and the reverse primer 5′-TCTTCCAGGGAACCCAGGAATGGCTGATCCGTTGA-3′ complementary to the vIII sequence and encoding a GS spacer. The PCR products were mixed together and the mixture was used to transform *E. coli* HK100 strain. After confirmation of the correctness of the gene fusion by sequence analysis, *E. coli* BL21DE3*ΔompA* strain was transformed with pET-Nm-fHbp-M30-vIII plasmid and the derived recombinant strain was used for the production of engineered M30-vIII-OMVs.

### Synthetic Peptides and Antibodies

The EGFRvIII peptide LEEKKGNYVVTDH unconjugated or conjugated to KLH protein was purchased from GeneScript in lyophilic form and solubilized in PBS at the final concentration of 1 mg/ml. Polyclonal antibodies against EGFRvIII peptide were obtained from GenScript by immunizing rabbits with KLH-conjugated LEEKKGNYVVTDH peptide.

The 27 amino acid M30 peptide PSKPSFQEFVDWENVSPELNSTDQPFL was purchased from GeneScript in lyophilic form and solubilized in milliQ water at final concentration of 5 mg/ml.

### Bacterial Total Lysate and OMV Preparation

Plasmids containing the genes of interest were used to transform *E. coli* BL21DE3*ΔompA* strain. Recombinant clones were grown in 200 ml LB medium (starting OD_600_ = 0.05) and, when the cultures reached an OD_600_ value of 0.5, protein expression was induced by addition of 1 mM IPTG. After 2 h, OMVs were collected from culture supernatants by filtration through a 0.22-µm pore size filter (Millipore) followed by high-speed centrifugation (200,000 *g* for 2 h). Pellets containing OMVs were finally re-suspended in PBS. Total bacterial lysates were prepared by suspending bacterial cells from 1 ml cultures (centrifuged at 13,000 *g* for 5 min) in sodium dodecyl sulfate-polyacrylamide gel electrophoresis (SDS-PAGE) Laemli buffer and heated at 100°C for 5 min. Proteins were separated by 4–12% or 10% SDS-PAGE (Invitrogen), run in MES buffer (Invitrogen), and finally stained with Coomassie Blue.

### Western Blot Analysis

Total lysates were prepared from bacteria grown in LB. Liquid cultures were pelleted in a bench-top centrifuge and suspended in SDS-PAGE loading buffer in an appropriate volume to normalize cell density to a final OD_600_ = 10. Each sample (10 µl) was then separated on a 4–12% SDS-polyacrylamide gel (Invitrogen). Proteins separated by SDS-PAGE were then transferred onto nitrocellulose membrane by standard methods. The membranes were blocked either 1 h at room temperature (RT) or overnight at 4°C by agitation in blocking solution (10% skimmed dry milk and 0.05% Tween 20 dissolved in PBS). Primary antibodies or sera were diluted in 1% skimmed dry milk plus 0.05% Tween 20 dissolved in PBS and incubated 1 h at RT. After three washing steps in 0.05% Tween 20 dissolved in PBS, the membranes were incubated in a 1:2,000 dilution of peroxidase-conjugated anti-rabbit or anti-mouse immunoglobulin (Dako) in 1% skimmed dry milk and 0.05% Tween 20 dissolved in PBS for 1 h, and after three washing steps, antibody binding was detected by using the SuperSignal West Pico chemiluminescent substrate (Pierce).

### Flow Cytometry Analysis

20 ml of LB medium supplemented with 100 µg/ml Ampicillin were inoculated at OD_600_ = 0.05 with an overnight culture of BL21*ΔompA*(pET-Nm-fHbp-vIII). The culture was then grown and IPTG-induced as described above. BL21*ΔompA*(pET21b+) strain was used as negative control. Bacterial cells from 1 ml were harvested by centrifugation at 10,000 *g* for 5 min at 4°C and re-suspended with 1% BSA in PBS to obtain a cell density of 2 × 10^7^ CFUs/ml. 50 µl were then dispensed in a round bottom 96 well plate. Anti-EGFRvIIII peptide rabbit antibodies were added at a concentration of 5 µg/ml and incubated 1 h on ice. After three washes with 1% BSA in PBS, 20 µl of FITC-labeled anti-rabbit secondary antibodies (1:200 dilution) (Life Technologies) were added and incubated 30 min on ice. Each well was then washed twice with 200 µl 1% BSA in PBS, and plates were centrifuged at 4,000 *g* for 5 min. Samples were then re-suspended in 2% formaldehyde solution, incubated 15 min at 4°C and centrifuged again at 4,000 *g* for 5 min. Finally, samples were re-suspended in 200 µl of PBS, and data were acquired by using BD FACS Canto II cell analyzer (BD).

### Confocal Microscopy Analysis

To verify fHbp-EGFRvIII localization on the cell surface, 20 ml of LB medium were inoculated at OD_600_ = 0.05 with an overnight culture of BL21*ΔompA*(pET-fHbpvIII). The culture was grown and protein expression induced with IPTG as described above. Bacterial cells from 1 ml culture were harvested by centrifugation at 6,000 *g* for 5 min at 4°C and re-suspended in 4% formaldehyde solution, incubated 15 min at 4°C and then centrifuged at 6,000 *g* for 5 min. Then samples were washed three times with 1 ml PBS, and incubated in 1 ml of blocking buffer (0.1% BSA, 10% normal goat serum in PBS) 20 min at RT. Subsequently, the bacterial suspension was incubated with rabbit polyclonal anti-EGFRvIIIpep antibodies (1 µg/ml) and mouse anti-LPS mAb (1 µg/ml) (Hycult Biotech, USA), for 1 h at RT. After two washes with 0.1% BSA in PBS, bacteria were incubated for 20 min at RT with goat anti-rabbit IgG, Alexa Fluor 594 conjugated-antibodies (Molecular Probes) and goat anti-mouse IgG, Alexa Fluor 488 conjugated-antibodies (Molecular Probes) at 1:400 final dilution. Labeled bacteria were washed twice with 0.1% BSA in PBS, and allowed to adhere to poly-lysine slides (Thermo Scientific) for 20 min at RT. Slides were mounted with ProLong Gold antifade reagent (Thermo Scientific). Confocal microscopy analysis was performed with a Leica SP5 microscope and images were obtained using Leica LASAF.

### TEM Analysis

Outer membrane vesicles purified from *E. coli* BL21*ΔompA*(pET-Nm-fHbpvIII) strain were visualized using Immuno TEM. Briefly, a 5-µl aliquot of purified OMVs preparation at a final concentration of 20 ng/µl was applied to 200-square mesh nickel grids coated with a thin carbon film (Agar Scientific) and let stand for 3 min. The samples were then blocked in 0.5% BSA in PBS for 1 h at RT. Subsequently, the samples were incubated with primary rabbit anti-EGFRvIIIpep antibodies for 1 h at RT. Grids were washed three times in blocking buffer and incubated with 5-nm gold-labeled anti-rabbit secondary antibody (BB International, Madison, WI, USA) for 1 h at RT. Immunostained OMVs were then negatively stained in 1% phosphotungstic acid and visualized with a Tecnai G2 Spirit Transmission Electron Microscope operating at 100 kV. Images were collected at 87,000× magnification with a CCD camera Morada 2kx4k.

### Vaccine Immunogenicity and Tumor Challenge in C57BL/6 Mice

#### Immunization with OMVs from BL21*ΔompA* (pET-Nm-fHbp-vIII) Strain

C57BL/6 mice (16 mice/group) were vaccinated on day 0, 14, and 28 with 20 µg of either “empty” OMVs [derived from BL21*ΔompA* (pET21b+) strain] or 20 µg of Nm-fHbpvIII-OMVs [derived from BL21*ΔompA*(pET-Nm-fHbp-vIII) strain] formulated in PBS. At day 35, 0.5 × 10^5^ B16F10EGFRvIII cells were subcutaneously (s.c.) injected in each animal and tumor growth was measured with a caliper every 3 days over a period of 30 days. For ethical reasons, mice were euthanized when tumors reached a size of 1,500 mm^3^.

#### Immunization with Nm-fHbp-vIII-OMVs Combined with B16-M30 Peptide

C57BL/6 mice (eight mice/group) were vaccinated on day 0, 14, and 28 with 20 µg “empty” OMVs, 20 µg Nm-fHbp-vIII-OMVs, 100 µg of synthetic B16-M30 peptide absorbed to 20 µg Nm-fHbp-vIII-OMVs, or 20 µg Nm-fHbp-M30-vIII-OMVs. At day 35, 0.5 × 10^5^ B16F10EGFRvIII cells were s.c. injected in each animal and tumor growth was followed as descried above.

#### Analysis of Anti-EGFRvIII Antibodies in Immunized Animals

Anti-EGFRvIII antibodies were measured by ELISA. Amino plates (Thermo Fisher) were coated with synthetic EGFRvIII peptide (0.5 µg/well) and incubated overnight at 4°C. The day after, plates were saturated with a solution of 1% BSA in PBS (200 µl per well) for 1 h at 37°C. Mice sera were threefold serially diluted in PBS supplemented with 0.05% tween (PBST) and 0.1% BSA. After three washes with PBST, 100 μl of each serum dilution were dispensed in plate wells. As positive control, Anti-EGFRvIII rabbit serum from animals immunized with KLH-conjugated LEEKKGNYVVTDH (EGFRvIII) peptide was used. After 2 h incubation at 37°C, wells were washed three times with PBST and then incubated 30 min at 37°C with mouse anti-rabbit alkaline phosphatase-conjugate antibodies at a final dilution of 1:2,000. After three washes with PBST, 100 µl of alkaline phosphatase substrate (Sigma Aldrich) were added to each well and plates were maintained at RT in the dark for 30 min. Finally absorbance was read at 405 nm using the M2 Spectramax Reader plate instrument.

#### T Cell Analysis

At the end of the tumor challenge studies described above (30 days from tumor cell administration) mice were sacrificed and spleens collected in 5 ml DMEM high glucose (GIBCO). Alternatively, mice were immunized twice i.p. at days 0 and 7 with either 20 µg “Empty” OMVs + 100 mg M30pep or 20 µg Nm-fHbp-M30-vIII-OMVs. Five days after the second immunization, mice were sacrificed and spleens collected. Spleens were then homogenized and splenocytes filtered using a Cell Strainer 70 µm. After centrifugation at 400 *g* for 7 min, splenocytes were re-suspended in PBS and aliquoted in a 96-well plate at a concentration of 1 × 10^6^ cells per well. Cells were stimulated with 2 mg/ml of an unrelated peptide (negative control), or 2 mg/ml of B16-M30 peptide. As positive control, cells were stimulated with phorbol 12-myristate 13-acetate (PMA, 0.5 mg/ml) and Ionomycin (1 mg/ml). After 2 h of stimulation at RT, Brefeldin A [Beckton Dickenson (BD)] was added to each well and cells incubated for 4 h at 37°C. After two washes with PBS, NearIRDead cell staining reaction mixture (Thermo Fisher) was incubated with the splenocytes for 20 min at RT in the dark. After two washes with PBS and permeabilization and fixing with Cytofix/Cytoperm (BD) using the manufacturer’s protocol, splenocytes were stained with a mix of the following fluorescent-labeled antibodies: anti CD3-APC (BioLegend), anti-CD4-BV510 (BioLegend), anti-CD8-PECF594 (BD), and IFN-γ-BV785 (BioLegend). Samples were analyzed on a BD FACSCanto II using FlowJo software. Graphs were processed with Prism 5.0 software (Graphpad). Statistical analysis and differences in means between two groups were calculated with a *t*-test calculator carried out using GraphPad Prism 5.03 (n.s.: *P* > 0.0.05, *P < 0.05, **P < 0.01, ***P < 0.001).

#### Analysis of TILs

Tumor-infiltrating lymphocytes were isolated from subcutaneous B16F10EGFRvIII tumors taken from sacrificed mice. At least two tumors per group were collected and minced into pieces of 1–2 mm of diameter using a sterile scalpel. Tumor samples were then transferred into 15-ml tubes containing 5 ml of collagenase solution (Collagenase Type 3,200 U/ml, Collagenase Type 4,200 U/ml) diluted in HBSS with 3 mM CaCl_2_ and incubated under agitation for 2 h at 37°C. The resulting cell suspensions were filtered through a Cell Strainer 70 µm, washed twice with PBS and 1 × 10^6^ cells were dispensed in a 96-well plate. Then, cells were incubated with NearIRDead cell staining Kit (Thermo Fisher) 20 min on ice in the dark. After two washes with PBS, samples were stained with the following mixture of fluorescent-labeled antibodies (BD): anti-GR1 (BV605), anti-CD11b-BV480, anti-CD45-BV786, anti-CD3-BV421, anti-CD4-PE, anti-CD8-PECF594, and anti-CD25-APC. The samples were then incubated 1 h at RT. After two washes with PBS, Cytofix/Cytoperm (BD) was added to each well and incubated 20 min on ice in the dark. After two washes with PBS, cells were stained with anti-Foxp3-A488 (BD) antibodies diluted in Permwash 1× buffer 20 min at RT in the dark. Finally, samples were washed two times with 1% BSA in PBS and analyzed on a BD FACSCanto II as described above.

## Ethics Statement

Mice were monitored twice per day to evaluate early signs of pain and distress, such as respiration rate, posture, and loss of weight (more than 20%) according to humane end points. Animals showing such conditions were anesthetized and subsequently sacrificed in accordance with experimental protocols, which were reviewed and approved by the Animal Ethical Committee of Toscana Life Sciences Foundation and by the Italian Ministry of Health.

## Author Contributions

GG: design of experimental plan and manuscript preparation. AG: experimental design, strain Engineering, analysis of immune responses, and animal studies. MP: analysis of Immune responses, FACS analysis, T cell analysis, and confocal microsacopy analysis. MT and IZ: animal studies. LG, EC, LFantappiè, SI, FZ, and CI: strain engineering and OMV preparation and characterization. LFagnocchi and EK: strain fermentation and OMV purification and characterization. ST and CS: FACS analysis of T cell population. FG and IF: electron microscopy analysis of OMVs.

## Conflict of Interest Statement

ST, CS, FG, and IF are employees of GSK (Siena, Italy). The Company has not financially supported the activities reported in the manuscript and has not expressed any commercial interest in the project.

## Supplementary Material

Figure S1 Flow cytometry gating strategy and analysis of tumor cell populations. Cells (1 × 10^6^) were isolated after collagenase treatment from frozen tumors collected from one mouse immunized with “Empty” OMVs **(A)** and one mouse immunized with Nm-fHbpvIII OMVs **(B)**. A first selection was made based on NearIRDead cell staining and only alive cells were included in the analysis. Subsequently, a homogeneous population of single cells was selected according to morphological parameters. The percentage of CD8+ and CD4+ T cells in each tumor was calculated from the CD45+/CD8+ and CD45+/CD4+ double positive cell populations, respectively. The double positive cells CD45+/CD4+ were subsequently selected for Treg analysis using anti-Foxp3+ antibodies. Finally, MDSCs were identified by selecting the CD45+ cell population and analyzing their positivity to CD11b and Gr1 staining.
