# Supplementary figures and images for: Synergistic Protective Activity of Tumor-Specific Epitopes Engineered in Bacterial Outer Membrane Vesicles
Source: Front Oncol. 2017 Nov 7;7:253. doi: 10.3389/fonc.2017.00253 (PMC5681935; doi:10.3389/fonc.2017.00253)

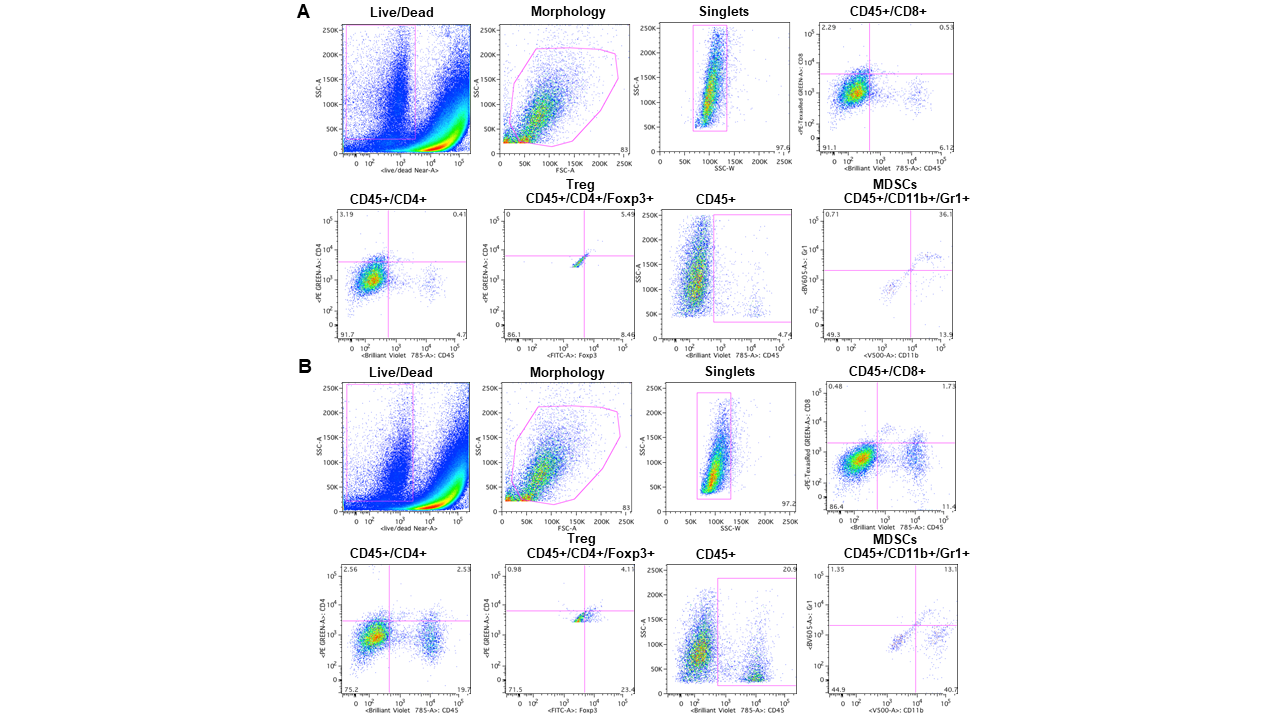

Supplement: Figure S1 — Flow cytometry gating strategy and analysis of tumor cell populations. Cells (1 × 10^6^) were isolated after collagenase treatment from frozen tumors collected from one mouse immunized with “Empty” OMVs (A) and one mouse immunized with Nm-fHbpvIII OMVs (B). A first selection was made based on NearIRDead cell staining and only alive cells were included in the analysis. Subsequently, a homogeneous population of single cells was selected according to morphological parameters. The percentage of CD8+ and CD4+ T cells in each tumor was calculated from the CD45+/CD8+ and CD45+/CD4+ double positive cell populations, respectively. The double positive cells CD45+/CD4+ were subsequently selected for Treg analysis using anti-Foxp3+ antibodies. Finally, MDSCs were identified by selecting the CD45+ cell population and analyzing their positivity to CD11b and Gr1 staining. [file image_1.tif]
